# Draft genome sequence of multi-drug resistant *Klebsiella quasipneumoniae* subsp. *similipneumoniae* isolated from a teaching hospital wastewater in South West, Nigeria

**DOI:** 10.1128/mra.00727-23

**Published:** 2024-01-25

**Authors:** Omowumi T. Akinola, Samuel O. Dahunsi, Anthony Okoh

**Affiliations:** 1 Microbiology Programme, College of Agriculture, Engineering, and Science, Bowen University Iwo, Iwo, Osun, Nigeria; 2 The Radcliffe Institute for Advanced Study, Harvard University, Cambridge, Massachusetts, USA; 3 SAMRC Microbial Water Quality Monitoring Centre, University of Fort Hare, Alice, South Africa; Loyola University Chicago, Chicago, Illinois, USA

**Keywords:** antibiotic resistance

## Abstract

This broadcast is about the whole genome sequence of *Klebsiella quasipneumoniae* subsp. *similipneumoniae* (ST 1422) isolated from a teaching hospital wastewater in South West, Nigeria, in May 2022. This data set compiles information on the DNA size (5,332,183 bp) and GC content (57.91%) in its genome.

## ANNOUNCEMENT

*Klebsiella* genus members are Gram-negative encapsulated non-motile bacteria that can acquire antibiotic resistance genes, causing mortality and morbidity (1). They cause both community- and hospital-acquired infections in humans. Pneumonia, upper respiratory tract infections, bacteremia, are some of these infections that affect people (2).

*Klebsiella quasipneumoniae* subsp. *similipneumoniae* is one of the member of the *Klebsiella pneumoniae* species complex (KpsC), identified on the basis of gyrA sequences and labeled as phylogroups of *K. pneumonia* (3). Due to the biochemical assays for *K. quasipneumoniae* and other *Klebsiella* spp. are identical, it is difficult to identify this organism using conventional laboratory techniques, which may result in misidentification and incorrect reporting (4), but whole-genome sequencing analysis can correctly identify it.

The sample was collected with a sterile bottle placed at hospital wastewater drain pool in Ogbomoso, Nigeria (8°.08′02.5″ N and 4°14′00.8″.23 E). The sample was filtered using the membrane filtering technique (Millipore, Merck, South Africa) via a 0.45-µm nitrocellulose filter membrane. The filter was placed on MacConkey agar plate and incubated at 37℃ for 24 h, after which pink mucoid colonies were subcultured to get a pure isolate. Biochemical test (5) results of indole negative, methyl red negative, and Voges-Proskauer positive revealed B105 to be *Klebsiella pneumonia*.

Genomic DNA was isolated from a pure culture plate using ZymoBIOMICS DNA Miniprep Kit (Zymo Research), following the manufacturers’ instructions. Using the custom-built IDT 10 bp unique dual indices with a desired insert size of 320 bp, Illumina sequencing libraries were created by means of the tagmentation- and PCR-based Illumina DNA Prep kit. In one or more multiplexed shared-flow-cell runs, the DNA was sequenced by Illumina on an Illumina NovaSeq 6000 sequencer, yielding 2 × 151 bp paired-end reads. The reads were trimmed for quality using Trimmomatic v0.36 (6).

*De novo* assembly of cleaned reads was performed with Read Annotation Assembly Pipeline Tool (RAPT v0.55teamcity CI) (https://github.com/ncbi/rapt). The assembled genome was annotated via Prokaryotic Genome Annotation Pipeline (PGAP) Table 1 (7). The assembled genome was submitted to the *Klebsiella* multilocus sequence type (MLST 2.0) database at the Center for Genomic Epidemiology using seven housekeeping genes (8).

**TABLE 1:** Features of B105 *Klebsiella quasipneumoniae* subsp. *similipneumoniae*

Features	Facts for B105: *Klebsiella quasipneumoniae* subsp. *similipneumoniae*
Sequence reads	
Total reads (bp)	1,462,203
Total length (bp)	5,332,183
Assembly	
DNA genome size (bp)	5,332,183
Contigs	96
Coverage (×)	81
G+C content (%)	57.91
Protein-coding genes	5,231
rRNA genes	8
tRNA genes	76
Contigs N50 (bp)	182,922
Contigs L50	10

K-mer inbuilt tool on the National Center for Biotechnology Information (NCBI) was employed to map sequence reads with the available sequences in database (9, 10). In the annotated result, strain B105 had an average nucleotide identity (ANI) of 99.185% with strain GCA_000613225.1, the type strain of *Klebsiella quasipneumoniae* subsp. *similipneumoniae* in the database.

## Data Availability

The BioProject accession number SAMN36174866 was used to store the sequencing data. The whole-genome shotgun of this project has been deposited at DDBJ/ENA/GenBank under the accession number JAUHJP000000000. The raw sequence reads have been deposited in the Sequence Read Archive (SRA) under accession number PRJNA989579.
